# Complex Mechanical Loading and Pro‐Inflammatory Cytokines in Intervertebral Disc Degeneration

**DOI:** 10.1002/jsp2.70159

**Published:** 2026-01-26

**Authors:** Katherine B. Crump, Estefano Muñoz‐Moya, Kim de Graaf, Paola Bermudez‐Lekerika, Nicole Schwendener, Wolf‐Dieter Zech, Christine L. Le Maitre, Jérôme Noailly, Benjamin Gantenbein

**Affiliations:** ^1^ Tissue Engineering for Orthopaedics & Mechanobiology (TOM), Bone & Joint Program, Department for BioMedical Research (DBMR), Faculty of Medicine University of Bern Bern Switzerland; ^2^ Graduate School for Cellular and Biomedical Sciences (GCB) University of Bern Bern Switzerland; ^3^ BCN MedTech, Department of Engineering Universitat Pompeu Fabra Barcelona Spain; ^4^ Institute of Forensic Medicine University of Bern Bern Switzerland; ^5^ Division of Clinical Medicine, School of Medicine and Population Health University of Sheffield Sheffield UK; ^6^ Department of Orthopaedic Surgery and Traumatology, Faculty of Medicine Inselspital, University of Bern Bern Switzerland

**Keywords:** catabolism, dynamic compression, finite element method, graph‐based network analysis, intervertebral disc, mechanobiology, organ culture, pro‐inflammatory cytokines, systems biology, torsion

## Abstract

**Background:**

Intervertebral disc (IVD) degeneration is a major contributor to low back pain, yet its initiating factors remain unclear. While the individual effects of pro‐inflammatory cytokines and mechanical loading on IVDs have been studied, their combined impact is poorly understood. This study investigated how dynamic compression and torsion interact with interleukin‐1 beta (IL‐1β) and its inhibitor, interleukin‐1 receptor antagonist (IL‐1Ra), using bovine IVDs in an ex vivo organ culture system.

**Methods:**

Whole bovine caudal IVDs were cultured for one week in a custom bioreactor applying diurnal dynamic compression (0.1–0.5 MPa) and torsion (±6°) under three media conditions: physiological, catabolic (10 ng/mL IL‐1β), and inhibitory (10 ng/mL IL‐1Ra). Static compression (0.1 MPa) served as control. 3 T magnetic resonance imaging (MRI) was used pre‐ and post‐culture for imaging and segmentation using 3DSlicer. Subject‐personalized finite element (FE) models were generated via morphing algorithms and coupled with a parallel network (PN) model to analyze metabolite transport and its impact on gene expression. Outcomes included disc height, glycosaminoglycan (GAG) content, qPCR, and cell metabolic activity.

**Results & Conclusions:**

Degenerative changes were detected in all treatment groups. Results of decreased disc height, hydration, and ACAN expression, alongside increased MMP‐13, indicated that the applied loading was supraphysiological and induced catabolic responses. IL‐1Ra, at the given dose, did not counteract degeneration. MRI‐based FE modeling effectively captured patterns of tissue consolidation and degeneration, providing valuable insights into IVD responses under combined mechanical and inflammatory stress. This integrative platform highlights the importance of modeling complex IVD environments and may inform the design of improved anti‐catabolic therapies.

## Graphical abstract

This study investigated the combined effects of dynamic compression and torsion with major pro‐catabolic cytokine interleukin‐1 beta (IL‐1β) and its inhibitor: interleukin‐1 receptor antagonist (IL‐1Ra), on bovine IVD in an ex vivo organ culture system and further evaluated the mechanical effects using a subject‐personalized finite element model based on MRI.

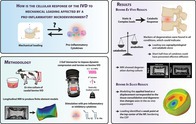

## 1 Introduction

Low back pain (LBP) is the leading cause of disability worldwide and is primarily due to intervertebral disc (IVD) degeneration [1, 2] Although degeneration is associated with altered tissue composition, inflammation, and biomechanical dysfunction, the initiating factors and their interactions remain poorly understood [1, 3, 4].

The IVD comprises a hydrated nucleus pulposus (NP), a collagen‐rich annulus fibrosus (AF), and thin cartilaginous endplates (CEP) [5, 6]. Together, these tissues maintain disc height and mechanical function through load‐dependent fluid movement and matrix turnover. In healthy discs, physiological mechanical loading supports extracellular matrix (ECM) homeostasis. However, abnormal or excessive loading can promote pro‐inflammatory signaling and matrix catabolism that promote degeneration. For example, elevated interleukin‐1β (IL‐1β) has been associated with progressive degeneration and can induce the expression of matrix‐degrading enzymes such as matrix metalloproteinases (MMPs) and a disintegrin and metalloproteinase with thrombospondin motifs (ADAMTS) [5, 7, 8, 9, 10]. Conversely, the endogenous IL‐1 receptor antagonist (IL‐1Ra) can counteract IL‐1β activity, but appears insufficiently upregulated in degenerated discs, making it of interest as a potential therapeutic mediator [9, 11, 12].

Dynamic mechanical loading also influences solute transport within the disc. Moreover, the combination of mechanical and pro‐inflammatory factors may further exacerbate disc degeneration, as dynamic loading can facilitate the transport of pro‐inflammatory cytokines [13] This can lead to a vicious cycle of degeneration and inflammation, further exacerbating the condition. Prior work has shown that exogenous cytokines, such as TNF, penetrate bovine IVDs under dynamic but not static conditions, suggesting that the mechanical environment regulates the exposure of resident cells to inflammatory cues [13] However, the interplay between IL‐1β or IL‐1Ra and complex mechanical loading in the form of combined compression and torsion has not yet been examined in whole‐organ IVD culture. However, at the time of publication, no studies have evaluated how IL‐1β or IL‐1Ra affects the cellular response to mechanical loading in the IVD, nor have any investigated how cytokines affect bovine IVDs under combined dynamic compression and torsional loading. As TNF, IL‐1β, and IL‐1Ra are similar in size [14] it was hypothesized that IL‐1β and IL‐1Ra would be able to transport through the IVD in a similar manner under dynamic but not static loading conditions. Additionally, it was hypothesized that IL‐1β in the IVD microenvironment is sufficient to negatively alter the cellular response to complex loading, leading to further degeneration, whereas stimulation with IL‐1Ra would be sufficient to prevent degeneration.

Further, the interpretation of mechanical and biological responses benefits from understanding how disc geometry evolves under load. Magnetic resonance imaging (MRI) enables non‐destructive monitoring of structural changes and facilitates the development of subject‐personalized finite element (FE) models to estimate internal mechanical and transport environments [15, 16, 17, 18] Integrating FE analysis with organ culture therefore offers an opportunity to link external loading conditions with internal multiphysics effects on protein activation in the presence of proinflammatory cytokines.

Therefore, the aim of this study was two‐fold: (1) To evaluate how IL‐1β and IL‐1Ra influence the cellular and matrix responses of bovine IVDs under ex vivo dynamic and static loading conditions, and (2) to develop MRI‐based, subject‐personalized FE models that can offer insights into loading‐dependent changes in disc geometry and internal multiphysics effects on protein activation over time. This combined experimental‐computational approach provides a novel approach into how mechanical loading and inflammatory signaling interact to drive IVD degeneration or, alternatively, to support disc homeostasis.

## 2 Methods

### 2.1 Bovine IVD Extraction

**FIGURE 1:**
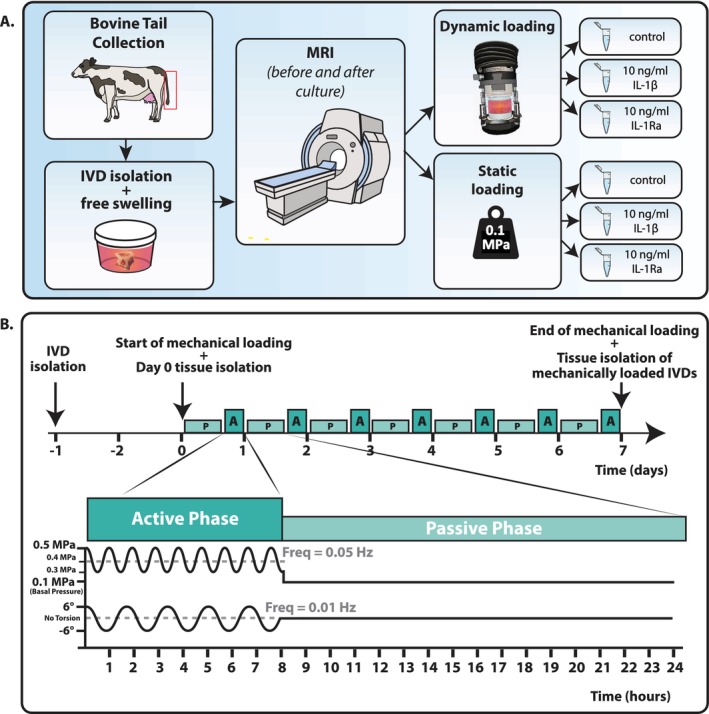
(A) Methodology of the experiment is shown. Bovine tails are first collected from an abattoir within 4 h of slaughter. 7–8 IVDs were then isolated from the tail and kept in free swelling for two days. Subsequently, 4–5 of the bovine IVDs were brought for imaging at the MRI, which was repeated after one week of culture. During culture, discs were loaded either dynamically or statically and treated with 10 ng/mL IL‐1β, IL‐1Ra, or left untreated as a control. (B) The dynamic loading regime consists of a passive phase of 0.1 MPa continuous compression for 16 h, followed by an active phase of 0.3–0.5 MPa dynamic compression in parallel with 6° torsion for eight hours. This cycle was then repeated six times for a total culture time of seven days.

Bovine tails were collected from a local abattoir within four hours of animal sacrifice (Figure 1A) to isolate their IVD for organ culture, as previously described by Chan et al. [14] Approximately 7 IVDs were isolated per biological replicate, that is tail. In brief, tails were immersed in a 1% Betadine solution for five minutes for disinfection and then transferred onto a sterile workstation. Muscle tissue surrounding the IVD was removed using a sterile scalpel blade. A custom‐made industrial blade holder was used to cut 1–2 mm away from the IVD towards the vertebrae. Any remaining growth plate was removed using a scalpel. The IVD was wrapped in sterile gauze presoaked in a solution of 0.9% sodium chloride and 55 mM sodium citrate solution to help clean the endplates from coagulated blood [14]. A caliper was used to measure the height and diameter of the isolated IVDs as previously described [19] which was used to determine the height and volumes of each IVD. The accuracy of this method has been determined previously with average intra‐observer error to be 1.69% for diameter measurement and 0.35% for disc height measurements. Following the measurements, each IVD was dipped into a 1% Betadine solution, and a Zimmer Pulsavac Plus jet lavage system was used to remove debris and blood clots from the IVDs with Ringer's Lactate solution. The IVDs were then washed twice for 5 min in 10X penicillin/streptomycin (1000 U/mL; #5711; Sigma‐Aldrich, Buchs, Switzerland), followed by a 5 min wash in phosphate‐buffered saline (PBS). Each IVD was then placed in a sterile container with 45 mL organ culture medium consisting of HG DMEM, 2% FBS, 1% penicillin/streptomyacin/glutamine (100 U/mL, 100 and 292 μg/mL, respectively; #10378–016; Thermo Fisher Scientific), 50 μg/mL ascorbic acid, 1% ITS+1, 10 mM sodium pyruvate, 1% HEPES buffer, and 1 μg/mg Amphotericin B. The containers were kept in an incubator (37°C, 5% CO_2_) at normoxia for two days, allowing free swelling of the IVDs during this period.

### 2.2 Bovine IVD Culture

After two days of free swelling to recover after IVD isolation, one of the IVDs was randomly selected as the day 0 sample and immediately processed, separating the NP, AF, and CEP tissues for downstream analysis. The remaining IVDs were assigned to either static or dynamic loading, either with or without the addition of 10 ng/mL of IL‐1β (Human IL‐1 beta Recombinant Protein # 200‐01B‐1MG, PeproTech) or 10 ng/mL of IL‐1Ra (Human IL‐1RA Recombinant Protein # 200‐01RA‐1MG, PeproTech), creating 6 different experimental groups (Figure 1 and Table 1).

**TABLE 1:** Overview of experimental conditions.

Regime	Loading parameters	Treatment
Day 0	*No load. Free swelling for 2 days*	*NA*
Static	*Continuous 0.1 MPa compression*	Control group
		10 ng/mL IL‐1β
		10 ng/mL IL‐1Ra
Dynamic	16 h passive phase: *continuous 0.1 MPa compression* 8 h active phase: *Dynamic 0.3–0.5 MPa compression and 6° torsion*	Control group
		10 ng/mL IL‐1β
		10 ng/mL IL‐1Ra

The static groups were subjected to a constant static load of 0.1 MPa, which is equivalent to pressure seen in a human lying prone [20]. The dynamic groups were subjected to a complex diurnal dynamic load in a bioreactor, which started on day 0 with a passive phase, in which a compressive force of 0.1 MPa was exerted onto the IVDs for 16 h. The passive phase was followed by an active phase of 8 h, during which dynamic compression was imposed, alternating between 0.3 and 0.5 MPa at a frequency of 0.1 Hz, and torsion of 6° at a frequency of 0.05 Hz simultaneously (Figure 1B). The dynamic compression was chosen to represent pressure in a human who is standing relaxed, while the torsion was chosen based on radiological measurements of maximum axial rotation [20, 21]. The alternation between the passive and active phases occurred continuously throughout the whole culture to mimic the physiologically mechanical loading of the spine [22]. Both the static and dynamic groups were cultured for 7 days at 37°C and 5% CO2 in normoxia. The culture medium was changed on days 2 and 5 of the culture with fresh organ culture medium, supplemented with 10 ng/mL IL‐1β or 10 ng/mL IL‐1Ra in the corresponding groups. At the end of the culture, each IVD was processed for downstream analysis and was split for RNA extraction and for GAG/DNA quantification. One biological replicate was taken for histological examination and protein extraction.

### 2.3 MRI Protocols

**FIGURE 2:**
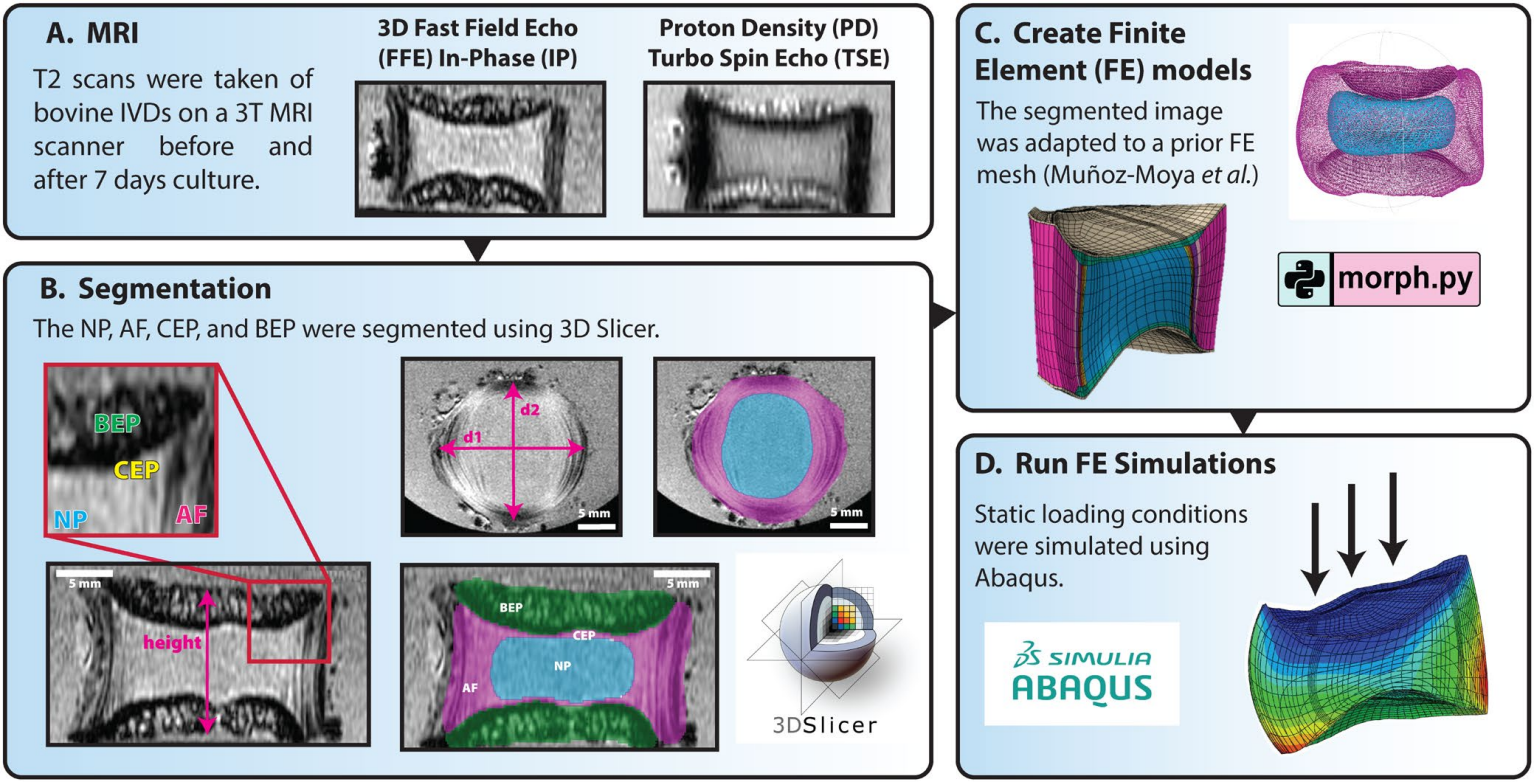
Methodology for developing a subject‐personalized FE model of bovine IVDs. (A) MRIs of the IVDs were taken on a 3 T MRI scanner using a proton‐density weighted (PD) turbo spin‐echo (TSE) sequence and a 3D fast field echo (FFE) in‐phase (IP) sequence. (B) 3D Slicer was then used to segment the NP, AF, CEP, and BEP from the MRI and produce an .stl file of the disc morphology. (C) Using a prior developed code [16], segmentations were adapted to fit an FE mesh. (D) Finally, boundary conditions were applied and the loading was simulated using Abaqus 2023.

During each organ culture, MRI scans were taken of the IVD on the day before the culture started and the same discs were scanned again on the final day of culture. Bovine IVDs were transported for imaging in a small plastic container in the same culture media as in the experiment (HG DMEM). Four to five conditions were scanned per biological replicate, which was alternated each replicate to ensure that all conditions were scanned at least once. (See Table S1) A 3 T Ingenia Elition X MRI scanner (Philips Healthcare, Best, the Netherlands) was used to make T2 scans of the IVDs using a proton‐density weighted (PD) turbo spin‐echo (TSE) sequence and a 3D fast field echo (FFE) in‐phase (IP) sequence (Figure 2A). Acquisition and spatial parameters of each sequence are presented in Table 2. Water content was estimated in the NP using mean signal intensity of the segmented NP region in the PD TSE scans relative to the signal intensity of the background. The signal intensity of the background was assumed to represent maximum (100%) water content as it was made up of the culture medium.

**TABLE 2:** MRI sequence parameters.

	Parameter	PD TSE	FFE
Acquisition parameters	Echo time (TE)	25 ms	6.365 ms
	Repetition time (TR)	1800	30
	Field of view (FOV)	16.2 × 15.5 mm	60 × 60 mm
	Number of averages	4	1
	Echo train length	7	1
	Pixel bandwidth	168	198
	Flip angle	90	15
Spatial parameters	Number of slices	23	81
	Voxel Size	0.0579 × 0.0579 × 0.5 mm	0.15 × 0.15 × 0.25 mm

### 2.4 FE Model

3D Slicer [23] was used to manually segment the NP, AF, CEP, and BEP (Figure 2A,B). The NP was depicted as the brighter section in PD TSE images, only slightly darker than the background, whereas the AF was depicted as dark (Figure 2A). The BEP appeared bright on PD TSE scans but dark on FFE scans (Figure 2A,B). The CEP was visualized as a bright line in between the NP or AF and BEP in FFE images (Figure 2B). The height and diameters of each disc were also measured using 3D Slicer (Figure 2B). Following segmentation, STL files were exported, and a previously published IVD morphing algorithm [16] was used to fit a generic FE mesh, previously validated and calibrated, to the bovine IVD and produce a final subject‐personalized bovine FE model for three of the statically loaded IVDs (Figure 2C). In brief, the algorithm exploits the Bayesian Point Cloud Drift (BCPD) technique and integrates an integrative morphing error control on the created elements and on the NP and AF shapes. Tissue material properties were those previously used by Muñoz‐Moya et al. [16] with the exception of water content and the initial fixed charge density, which were set to experimentally measured values and collagen content, which was taken from prior studies [24] (Table 3). Fixed charge density was calculated as previously described by Wills et al. using the valency (2 mEq/mmol), the molecular weight (513 000 μg/mmol), and the concentration (in μg/mL) of chondroitin sulphate [25] The concentration of chondroitin sulphate was determined from experimental GAG content normalized to water content in sample.

**TABLE 3:** Overview of FE model boundary conditions.

Bovine IVD	Displacement	Initial water content	Collagen content	Initial glucose	Initial lactate	Initial pH	Initial fixed charge density level
Control static	2.384 mm	78.97%	*NP:* 375 mg/g *AF:* 690 mg/g	5 mM	0	7.4	*NP:* 0.199 mEq/mL *AF:* 0.095 mEq/mL
IL‐1β static	2.57 mm	79.70%	*NP:* 375 mg/g *AF:* 690 mg/g	5 mM	0	7.4	*NP:* 0.219 mEq/mL *AF:* 0.045 mEq/mL
IL‐1Ra static	3.18 mm	73.90%	*NP:* 375 mg/g *AF:* 690 mg/g	5 mM	0	7.4	*NP:* 0.144 mEq/mL *AF:* 0.033 mEq/mL

Simulations were run using Abaqus 2023 (Figure 2D) [26]. Three statically loaded bovine IVDs (one control, one treated with IL‐1β, and one treated with IL‐1Ra) were modeled separately with their respective material properties (Table 3). Boundary conditions included external atmospheric pressure and zero displacements at the bottom of the disc. Simulations began with 17 h of free swelling to reach the steady state between internal and external pressures. A vertical displacement was applied on the top of the IVD to match the corresponding experimental 7‐day height loss: 2.384 mm, 2.57 mm, and 3.18 mm, respectively (Table 4). This displacement was applied over six days using a ramp function (from zero to the imposed displacement) and maintained at the imposed displacement for 24 h for seven days. The subject‐personalized FE models were used to predict the transport of oxygen, glucose, lactate, and pH. They were then coupled with the PN equations previously published by Baumgartner et al. [27, 28] to calculate mRNA expression (*ACAN, COL1A1, COL2A1, and MMP‐3)*. mRNA expression was calculated at two time points per disc: after swelling and after the mechanical and transport steady state at 168 h (7 days). At the second time point, glucose, pH, and mechanical cell stress values were measured at the end of day 7, and the PN equations were evaluated with a time dependency of 168 h. This was performed at each integration point of the NP finite element, enabling localized predictions of mRNA response.

**TABLE 4:** Water content in the NP after swelling and creep.

Treatment	Source	Average water content%		Average glucose level (mM)	Average pH	Average cell stress (MPa)
		Day 0	Day 7			
Control	Experiment	79.0%	75.7%	—	—	—
	FE Model	78.3%	72.8%	3.57	7.23	0.54
IL‐1β	Experiment	79.7%	75.5%	—	—	—
	FE Model	78.9%	73.5%	3.63	7.24	0.57
IL‐1Ra	Experiment	73.9%	76.8%	—	—	—
	FE Model	74.9%	62.7%	3.51	7.22	1.25

*Note:* Experiment values are derived from mean signal intensities of the segmented NP region from the MRI PD‐TSE scans as reported in Figure 3D. The FE model values are initally set to those measured in the corresponding experimental IVD, but vary slightly due to the initial free swelling period simulated in the model.

This allowed comparison of expression before and after static compression over the 7 days. The predicted expression after compression was normalized to the expression after swelling, thereby reproducing the experimental normalization to day 0 as a perturbation of the steady state induced by mechanical stimuli. Predictions of gene expression from the simulated IVDs were directly compared with gene expression measured by qPCR in the corresponding IVD.

### 2.5 Cell Metabolic Activity

Cell metabolic activity of NP and AF tissues was assessed using a resazurin sodium salt solution. Resazurin is reduced to resorufin by metabolically active cells; this reduction results in a change in fluorescence [29] Immediately following tissue isolation, the tissue samples were weighed and transferred to a 48 wells plate, where 500 μL of 50 μM resazurin sodium salt solution was added to each well. After 1.5 h of incubation in the dark at 37°C and 5% CO_2_, fluorescence was measured using an ELISA reader (Spectramax M5, Molecular Devices, Bucher Biotec AG, Basel, Switzerland) with excitation at 540 nm and emission at 590 nm. Tissues were then washed with 1xPBS and dried overnight at 60°C. The following morning, samples were weighed and placed in 1 mL per 100 mg wet weight papain solution (3.9 U/mL papain (Sigma‐Aldrich; #P‐3125) and 5 mM L‐cysteine hydrochloride (Sigma‐Aldrich; #1161509)). Tissues were digested overnight at 60°C and stored at −20°C until DNA and GAG content were determined.

### 2.6 GAG and DNA Quantification

The GAG content in the tissue following papain digestion was determined using 1,9‐dimethyl‐ methylene blue (Sigma‐Aldrich; #341088) [23]. Absorbance was measured at a wavelength of 600 nm and results were interpolated using a standard curve based on chondroitin sulphate (Sigma‐Aldrich; #C6737).

The DNA content was measured from digested tissue using Hoechst 33258 dye (#86d1405; Sigma‐Aldrich) [22]. The optical fluorescence index was measured at 350 nm excitation and 450 nm emission wavelengths in digested samples and results were interpolated using a standard curve based on increasing DNA sodium salt concentrations from calf thymus (#D1501; Sigma‐Aldrich).

### 2.7 RNA Isolation and qPCR Analysis

On days 0 and 7 of the culture, isolated NP, AF, and CEP tissues from the IVDs were immediately snap‐frozen in liquid nitrogen and stored at −80°C. Upon use, the tissues were crushed in a precooled mortar, transferred to 1 mL TRIzol reagent (Life Technologies), and homogenized using a tissue lyser. RNA was extracted from samples using the GenEluteTM Mammalian Total RNA Miniprep kit (Sigma‐Aldrich; #RTN70‐1KT), in accordance with the manufacturer's protocol. Genomic DNA was digested using on‐column DNase I (Sigma‐Aldrich; #DNASE70‐1SET). RNA was then retrotranscribed into complementary DNA (cDNA) using the High‐Capacity cDNA kit (Thermo Fisher Scientific; #4368814) on a MyCycler Thermal Cycler (Bio‐Rad; #1709703).

For subsequent qPCR, the cDNA was mixed with the selected relevant primers (see Table 5) and iTaq Universal SYBR Green Supermix (Bio‐Rad; #1725122). Finally, qPCR was performed using a CFX96 Real‐Time System (Bio‐Rad; #1855096). Two technical replicates of each sample were averaged, and the mean was used for subsequent analysis. Relative gene expression was determined using the 2^−ΔCt^ method, with normalization to the 18S reference gene [30]. Undetected expression was set to the highest cycle number for analysis. Samples with 18S expression appearing at a cycle greater than 20 were excluded due to low‐quality RNA.

**TABLE 5:** qPCR primer sequences.

Gene Type	Gene (Gene Abbreviation)	Gene ID	Primer sequence Primer Sequence
Reference gene	18S	*18S*	f—ACG GAC AGG ATT GAC AGA TTG r—CCA GAG TCT CGT TCG TTA TCG
Anabolic markers	Aggrecan	*ACAN*	f—GGC ATC GTG TTC CAT TAC AG r—ACT CGT CCT TGT CTC CAT AG
	Type I collagen	*COL1A2*	f—GCC TCG CTC ACC AAC TTC r—AGT AAC CAC TGC TCC ATT CTG
	Type II collagen	*COL2A1*	f—CGG GTG AAC GTG GAG AGA CA r—GTC CAG GGT TGC CAT TGG AG
Catabolic markers	Matrix metalloproteinase‐3	*MMP‐3*	f—CTT CCG ATT CTG CTG TTG CTA TG r—ATG GTG TCT TCC TTG TCC CTT G
	Matrix metalloproteinase‐13	*MMP‐13*	f—CTT CCG ATT CTG CTG TTG CTA TG r—ATG GTG TCT TCC TTG TCC CTT G

### 2.8 Histology

IVDs were cut in half on the sagittal plane and fixed in 4% formaldehyde for 48 h. After fixation, the samples were washed in running tap water and decalcified for 4 months at RT in 12.5% EDTA, with EDTA changed 1–2 times per week. Following decalcification, samples were rinsed, dehydrated using a histokinette (Myr STP‐120), and embedded in paraffin. The paraffin‐embedded sections were cut into 4–7 μm thickness using a microtome and mounted on Superfrost Plus slides (ThermoFisher Scientific, Basel, Switzerland). The sections were deparaffinized, stained with Alcian Blue, or processed for immunohistochemistry as described below. For Alcian Blue staining, slides were acidified with 3% (v/v) acetic acid for 3 min, then incubated in 1% (v/v) Alcian Blue in 3% acetic acid (pH 1) for 30 min at RT. After rinsing in distilled water, slides were washed in distilled water for 10 min, then dehydrated in graded ethanol and cleared in xylene (all materials from Sigma‐Aldrich, Buchs, Switzerland). Finally, slides were mounted with Eukitt (Bio‐Optica Milano inc., Milano, Italy). Slides were imaged using a PANNORAMIC 250 Flash II DX slide scanner (3DHistech) microscope in brightfield at 20× magnification (Sysmex, inc., Etten‐Leur, The Netherlands).

### 2.9 Immunohistochemistry

Immunohistochemical staining of human IL‐1β (M421B, ThermoFisher Scientific, inc., 1:400 dilution) and human IL‐1Ra (ab124962, abcam, 1:4000 dilution) was performed following previously established protocols [31] using the VECTASTAIN Elite ABC Universal PLUS Kit Peroxidase (Horse Anti‐Mouse/Rabbit IgG) kit (Lubioscience limited, Lucerne, Switzerland). After deparaffinization and rehydration, endogenous peroxidases were blocked for 30 min in the BLOXALL blocking solution (VECTASTAIN Elite ABC Universal PLUS Kit Peroxidase). Antigen retrieval was performed using enzymatic retrieval [0.1% (w/v) α‐chymotrypsin (Sigma Aldrich, Poole, UK) in tris‐buffered saline (20 mmol/L Tris, 150 mmol/L NaCl, pH 7.5) containing 0.1% (w/v) CaCl2 for 30 min at 37°C]. After antigen retrieval, sections were rinsed in tris‐buffered saline (TBS, (20 mmol/L Tris, 150 mmol/L NaCl pH 7.5)) and blocked for 1 h at room temperature with 1% (w/v) bovine serum albumin (BSA) solution containing 25% (v/v) normal goat serum in TBS or with normal horse serum 2.5% (VECTASTAIN Elite ABC Universal PLUS Kit Peroxidase) to prevent nonspecific antibody binding. Primary antibodies were incubated overnight at 4°C in TBS with 1% (w/v) BSA. Simultaneous IgG control experiments were performed using the same protein concentrations to evaluate nonspecific isotype binding. (See Figure S1) After incubation, sections were rinsed in TBS four times, followed by the addition of secondary antibodies at room temperature for 30 min. After three more TBS washes, EliteABC reagent (VECTASTAIN Elite ABC Universal PLUS Kit Peroxidase) was applied for 30 min. This was followed by an additional four TBS washes before the addition of ImmPACT DAB EqV Reagent 1 (Chromogen) in ImmPACT DAB EqV Reagent 2 (Diluent) for 20 min, followed by 5 min of running tap water rinses. The nuclei were counterstained with Gill's Hematoxylin for 7 min and differentiated in 0.5% HCL followed by bluing under running water for 5 min. After dehydration in graded ethanol and clearing in xylene, the slides were mounted with Eukitt. Slides were scanned at 20× magnification using a PANNORAMIC 250 Flash II DX slide scanner (3DHistech), and representative images were captured to illustrate immunohistochemical staining. Immunopositivity was quantified using a previously developed semiautomated QuPath‐based script [32].

### 2.10 Statistical Analysis

All statistical analyses of gene expression, GAG and DNA quantification, cell metabolism, height, volume, and water content were performed under the assumption of a nonparametric distribution. Thus, a Kruskal‐Wallis test followed by a Dunn's multiple comparisons *post hoc* test was used for the evaluation of results. A Benjamini‐Hochberg correction was applied to account for multiple comparisons across the factors of treatment, loading, and time. Results were log‐transformed prior to statistical analysis. All statistical analyses were performed using R (R Core Team, 2020) and RStudio (R Version 4.4.0, R Studio Team, 2020), specifically the R stats and dunn.test packages [33]. A *p*‐value < 0.05 was considered statistically significant. All quantitative results are presented as median up to six biological replicates, and the exact number of biological replicates (n) is indicated in each figure legend.

## 3 Results

### 3.1 Anabolic Gene Expression Was Decreased in the NP After 7 Days Culture

To determine the cellular response to IL‐1β or IL‐1Ra treatment and static or dynamic loading, qPCR was performed to quantify gene expression in key anabolic and catabolic markers in the NP, AF, and CEP. While gene expression was not significantly different between groups, several genes were altered in relation to their initial expression on day 0 (Figure 4).

After seven days culture, expression of anabolic genes *ACAN* significantly decreased (*p* < 0.05) in the NP of all conditions except for the statically loaded control (*p* = 0.1001) and dynamically loaded, IL‐1Ra‐treated discs (*p* = 0.088). Similarly, *ACAN* was significantly decreased in the CEP for all conditions (*p* < 0.05), excluding the IL‐1Ra‐treated IVDs, where the decrease was a trend (*p* = 0.055). Interestingly, gene expression of *COL2A1, MMP3*, and *MMP13* was found to be significantly different in the AF relative to day 0. Specifically, *COL2A1* was significantly decreased in the IL‐1β‐treated groups (*p* < 0.05), *MMP3* was significantly decreased in the control groups (*p* < 0.05), and *MMP13* was significantly elevated in all conditions (*p* < 0.001, except for the control static where *p* = 0.002). Gene expression of *MMP13* in the NP trended towards an increase relative to day 0 in all conditions (*p* = 0.086) except for the statically loaded, IL‐1Ra‐treated discs. Similarly, *MMP13* expression trended towards a decrease in the CEP (*p* < 0.15) across all conditions except the dynamically loaded, IL‐1Ra‐treated discs. *COL1A2* expression was not affected in any culture condition. *ADAMTS4* and *IL‐1β* gene expression did not differ significantly across conditions (see Figure S2).

### 3.2 Height and Volume Were Decreased After Culture in All Conditions

**FIGURE 3:**
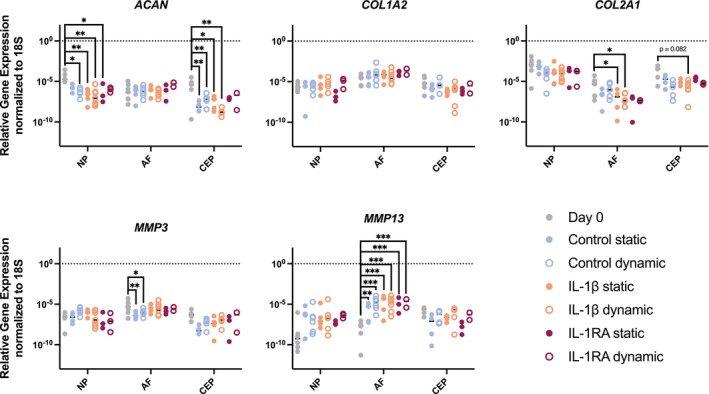
Measured (A) height and (B) volume differences in bovine IVDs after seven days of culture in the bioreactor or with static loading relative to day 0. (C) Measured water content in the NP and AF tissue at day 0 and after seven days of culture in the bioreactor or with static loading. (D) MRI‐derived water content based on signal intensities in PD TSE scans. Shown are the medians, *n* = 4–5.

Height and volume of the bovine IVDs decreased in all conditions compared to their initial height or volume at day 0, more so in dynamically loaded IVDs compared to statically loaded IVDs (Figure 3). Control statically loaded IVD heights decreased between 7.1% and 19.7% (average 14%), while dynamically loaded IVD heights decreased significantly more, ranging from 21% to 30.9% (average 26%) height loss (*p* = 0.011) (Figure 3A). Similarly, IL‐1β treated statically loaded discs decreased between 7% and 25.5% (average 17.2%), while dynamically loaded IVD heights decreased significantly more, ranging from 27.1% to 33.1% (average 30.3%) height loss (*p* = 0.003) (Figure 3A). Interestingly, height changes after culture were not significantly different between static (19.5% average decrease) and dynamically loaded (25.6% decrease) IVDs which were treated with IL‐1Ra (*p* = 0.143) (Figure 3A).

**FIGURE 4:**
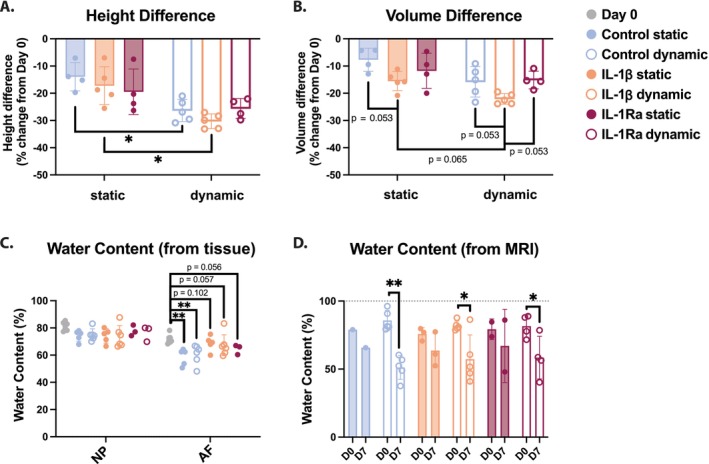
Relative gene expression of ACAN, COL1A2, COL2A1, MMP‐13, and MMP‐3 in the NP, AF, and CEP after culture. Data were normalized to 18S using the 2‐^ΔCt^ method. All data points are shown with medians, *n* = 1–6.

**FIGURE 5:**
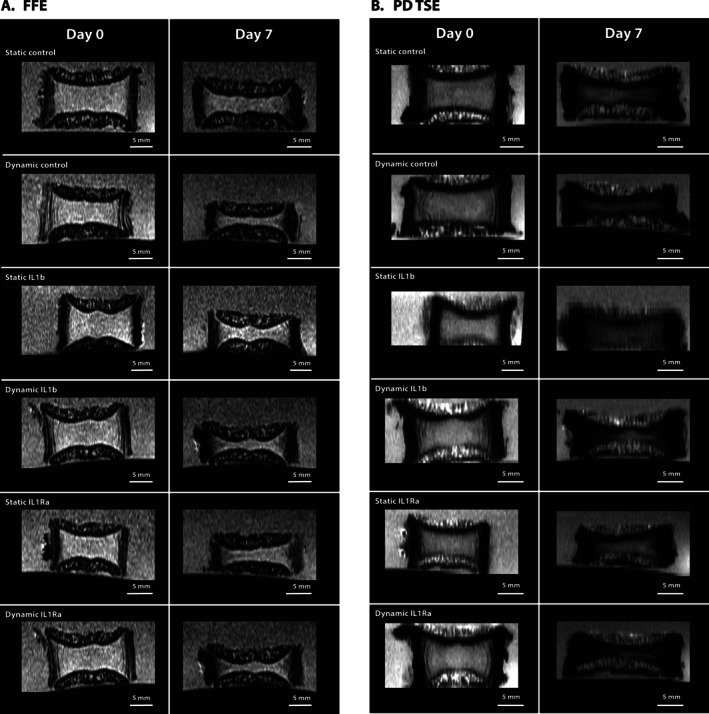
(A) Longitudinal fast field echo (FFE) MRI scans of bovine IVDs on day 0 and after seven days culture. (B) Longitudinal proton density‐weighted turbo spin echo (PD‐TSE) MRI scans of bovine IVDs on day 0 and after seven days of culture. Scale bars show 5 mm length.

**FIGURE 6:**
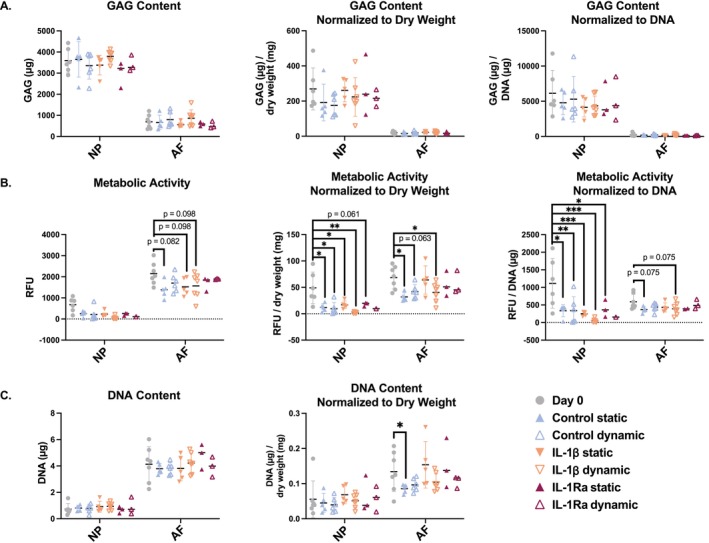
(A) Glycosaminoglycan (GAG) content in the NP and AF was measured on day 0 and after seven days of culture in the bioreactor or with static loading. Values shown are μg GAG content (left), GAG content normalized to dry weight (middle), and GAG content normalized to DNA (right). (B) Cell metabolic activity was measured in the NP and AF on day 0 and after 7 days of culture in the bioreactor or with static loading. Values shown are raw metabolic activity readout (left), metabolic activity normalized to dry weight (middle), and metabolic activity normalized to DNA (right). (C) DNA content was measured in the NP and AF on day 0 and after culture. Values shown are μg DNA (left) and DNA normalized to dry weight (middle). Shown are the medians, *n* = 3–7.

**FIGURE 7:**
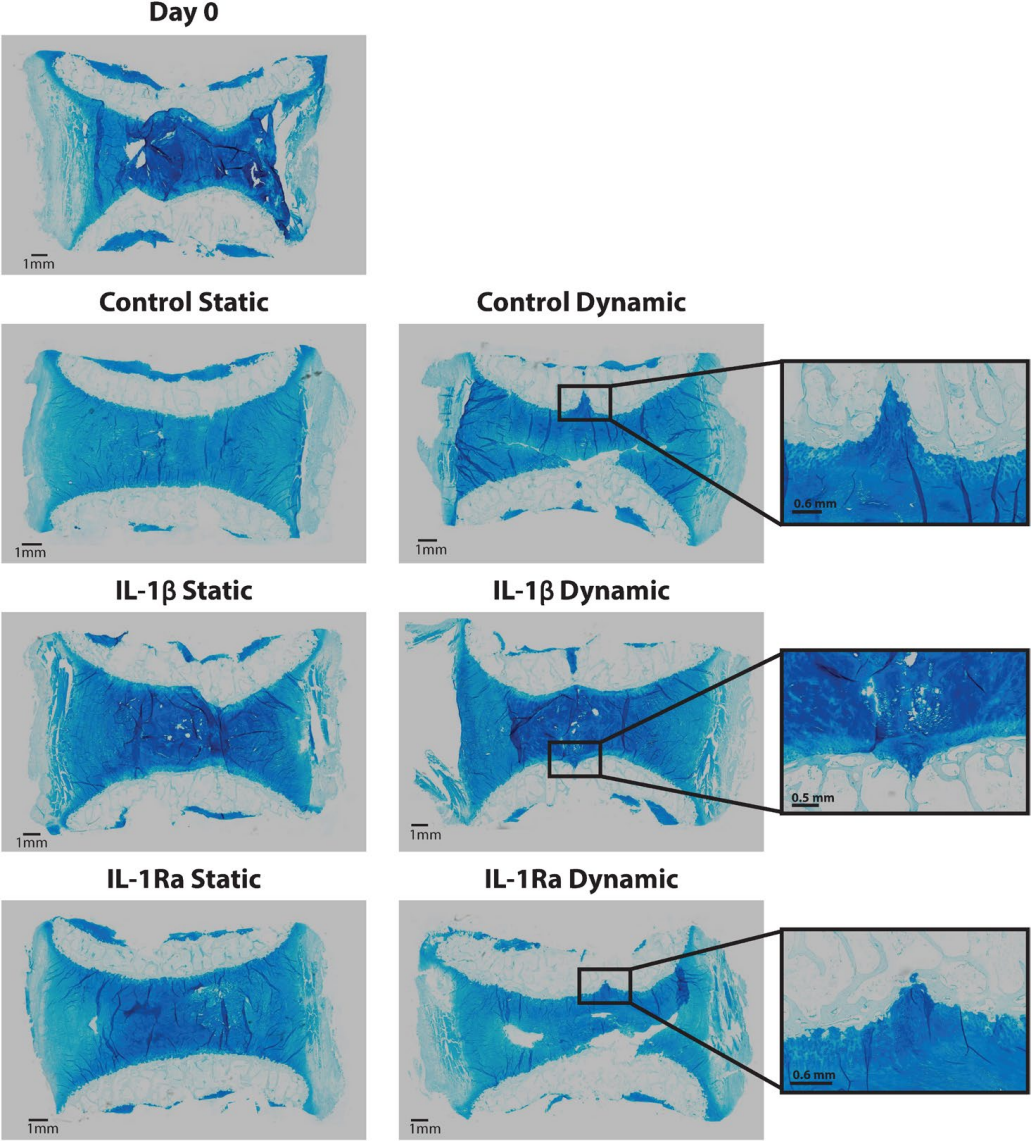
Alcian blue histological staining of bovine IVDs for each condition on day 0 and after 7 days of culture in the bioreactor or with static loading. Zoomed‐in panels on the right show regions of NP bulging after dynamic loading. Scale bars indicate 1 mm (0.8 mm in the zoomed‐in areas); *n* = 1.

**FIGURE 8:**
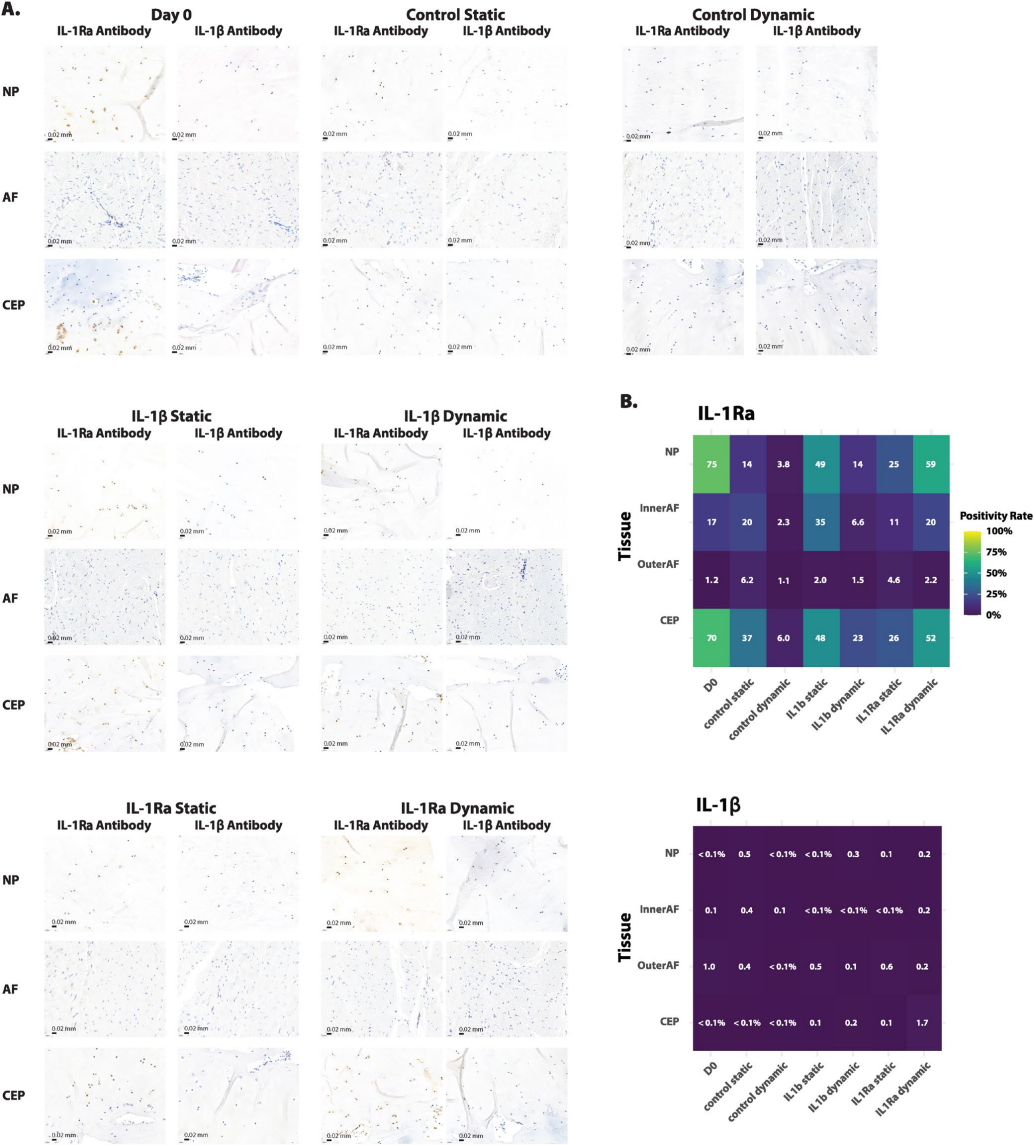
(A) Immunohistochemical staining for the presence of human IL‐1β (left) and IL‐1Ra (right) in the NP, AF, and CEP of bovine discs before and after 7 days of culture. Brown indicated the detection of the target antibody. Nuclei are counterstained with hematoxylin, shown in blue. The scale shows 0.02 mm. (B) Immunopositivity of IL‐1β (left) and IL‐1Ra (right) of cells in the NP, inner and outer AF, and CEP in bovine IVD tissue through IHC analysis quantified using QuPath. [32], *n* = 1.

**FIGURE 9:**
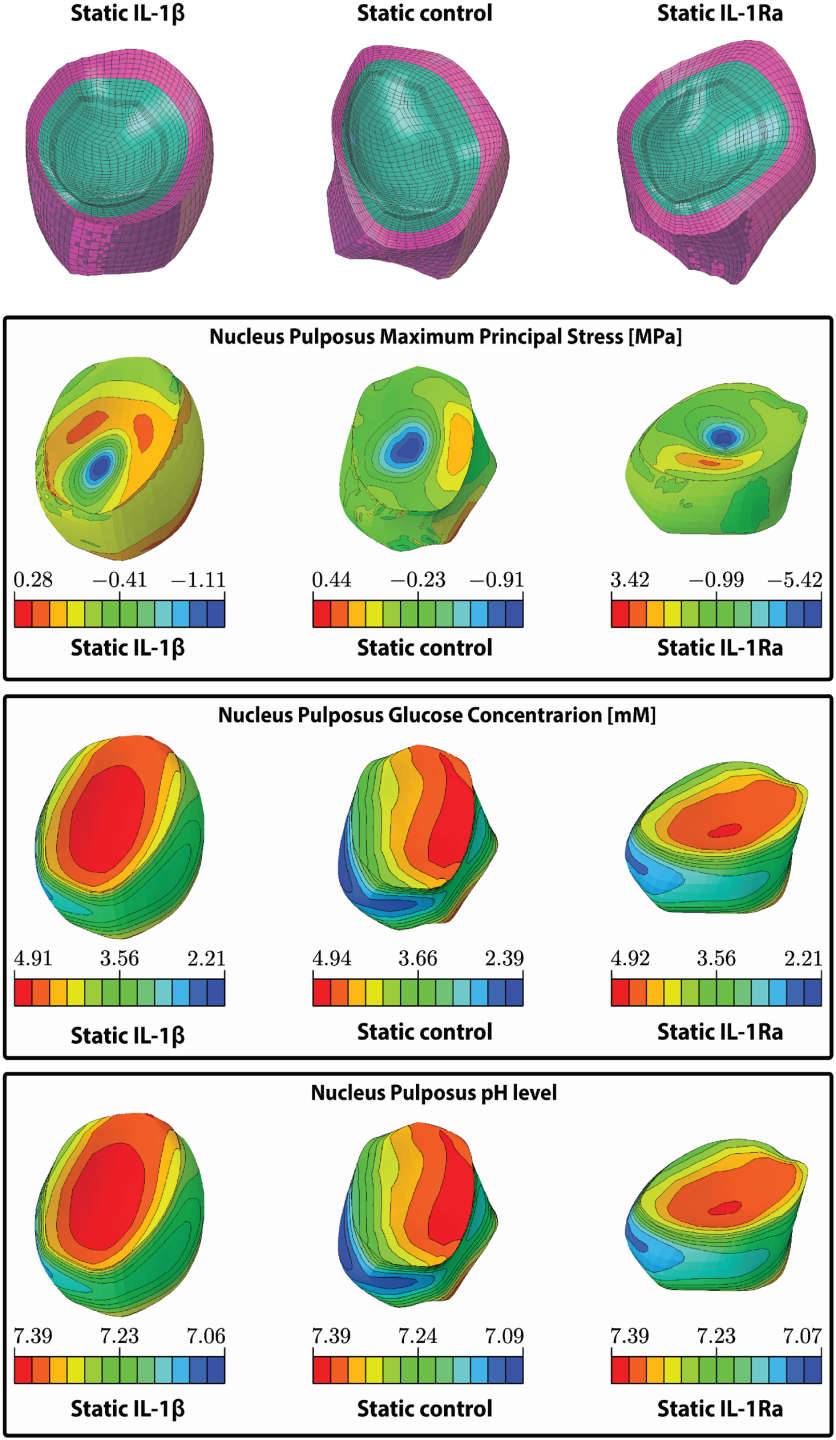
Mechano‐Transport FE simulation results. (A) The subject‐personalized FE models of the static control‐, IL‐1β‐, and IL‐1Ra‐treated bovine IVDs. (B) The predicted maximum principal stress (MPa) in the NP of each IVD. (C) The predicted glucose concentration (mM) within the NP for each IVD. (D) The predicted pH level in the NP of each IVD.

**FIGURE 10:**
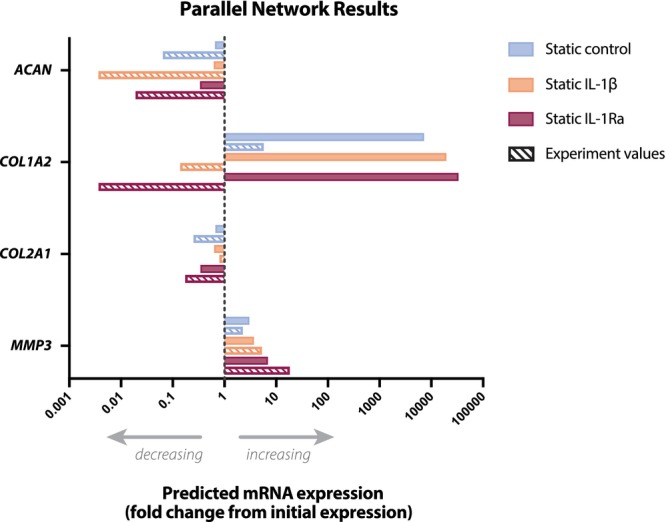
Parallel network (PN) simulation results compared to experimental gene expression values. Predicted mRNA expression is shown in solid color, while gene expression data is shown as striped. All values are relative to the initial values at day 0 (experiment) or after simulated swelling (PN).

While volume decreased in all conditions, the difference was not as high as the change in height (Figure 3B). Statically loaded IVD volumes decreased an average of 7.7% in controls, 15.6% in IL‐1β treated discs, and 11.8% in IL‐1Ra treated discs (Figure 3B). Dynamically loaded IVD volumes decreased an average of 15.8% in controls, 22.1% in IL‐1β treated discs, and 15.2% in IL‐1Ra treated discs (Figure 3B). The reduction in volume was not significantly different between any conditions; however, there was a trend that in dynamically loaded IVDs, IL‐1β treated IVD volumes decreased more than those of controls (*p* = 0.053) and IL‐1Ra treated IVDs (*p* = 0.053) (Figure 3B). Similarly, in statically loaded IVDs, IL‐1β treated IVD volumes trended towards a decrease in comparison to control IVD volumes (*p* = 0.053) (Figure 3B).

### 3.3 Water Content Was Decreased After Culture

Based on tissue measurements, water contents relative to day 0 in the NP were not significantly different from each other when compared across the different culture conditions (Figure 3C). However, water content in the NP was less in all conditions compared to the water content measured at day 0. While the average initial water content was 80.8% ± 3%, the NPs of control static and dynamically loaded discs had average water contents of 74% ±% 4% and 73.9% ± 4.4%, respectively. Water content in the NPs of IL‐1β‐treated static and dynamically loaded discs was 73.9% ± 5.3% and 67.5% ± 17%, respectively. The NP of IL‐1Ra treated static and dynamically loaded discs consisted of 77.5% ± 4% and 76.2% ± 5.7% water, respectively. Interestingly, water content did significantly decrease in the AF of control static and dynamically loaded IVDs relative to day 0 (*p* = 0.001). Additionally, water content in the AF of IL‐1β‐treated dynamically IVDs was significantly decreased relative to water content at day 0 from 71.5% ± 3.3% to 67% ± 7.7% water (*p* = 0.018).

### 3.4 MRI Conveys Disc Height and Loss of Hydration in All Conditions After Culture

Both MRI sequences showed the loss of disc height in all conditions, alongside the decreased size of the NP (Figure 5). Water content of the NP was also evaluated using the PD TSE scans from the MRI, providing corresponding values for the same IVD at day 0 and on day 7 (Figure 3D). Notably, water content in the NP was only significantly decreased in dynamically loaded discs (*p* < 0.05 for IL‐1β and IL‐1Ra treated IVDs and *p* < 0.01 for control IVDs), and not in statically loaded discs. No significant differences in water content were seen due to loading or cytokine treatment.

### 3.5 Metabolic Activity Was Decreased While GAG Content Remains Constant

Cell metabolic activity was significantly decreased in the NP in all conditions when normalized to the respective dry weight or DNA content (Figure 6B). All conditions were decreased considerably relative to metabolic activity in the NP at day 0. In particular, the metabolic activity of the NP under IL‐1β‐treated static and dynamic loading conditions decreased by 52% and 94%, respectively, relative to day 0 (*p* < 0.001). Metabolic activity of control static and dynamically loaded conditions in the NP was also significantly reduced relative to day 0 (*p* = 0.005 and *p* = 0.012, respectively). Regarding IL‐1Ra‐treated conditions, the statically loaded NP metabolic activity was significantly decreased relative to day 0 (*p* = 0.038). However, technical issues processing NP tissue led to insufficient values for statistical analysis for the dynamically loaded specimens.

Overall, GAG production did not differ significantly between NP and AF after 7 days of culture across conditions (Figure 6A). The measured content was normalized to the dry weight and the DNA content of the respective isolated tissue. GAG content remained relatively constant in both the NP and the AF throughout the experiment. DNA content remained relatively steady throughout the experiment, and no significant differences were observed across conditions. However, when normalized to the dry weight, the DNA content of control statically loaded AF was significantly decreased in comparison to the initial DNA content of the AF at day 0 (Figure 6C).

### 3.6 Alcian Showed NP Bulging Under Dynamic Compression

Alcian blue staining showed similar proteoglycan staining in all conditions; however, staining demonstrated that the nucleus bulged into the endplate for all dynamically compressed discs, but not static discs (Figure 7). This pattern was also seen in the corresponding MRI of dynamically loaded discs on day 7. Additionally, each bovine IVD showed some remnants of the growth plate attached to the outer BEP.

### 3.7 Human IL‐1Ra, but Not IL‐1β Was Detected in the NP of Bovine IVDs

Immunohistochemical staining was performed on statically and dynamically loaded bovine IVDs to test the presence of human IL‐1Ra and human IL‐1β before and after culture for one biological replicate (Figure 8A). As analysis was based on only one biological replicate, all data are preliminary and additional replicates are needed to draw any conclusions. Notably, NP cells were positive for IL‐1Ra at day 0 and in all culture conditions (Figure 8B). However, immunopositivity was highest at day 0 (75%) followed by the IL‐1Ra‐treated, dynamically compressed IVDs (59%) and the IL‐1β treated, statically loaded discs (49%). Interestingly, immunopositivity for IL‐1Ra was less apparent in control discs (14% and 4% in static and dynamically loaded IVDs, respectively) compared to day 0 or IL‐1β or IL‐1Ra treated discs. Cells in the inner AF showed between 2% and 35% immunopositivity, less than in the NP except for in the control static condition. In contrast, outer AF cells were rarely immunopositive for IL‐1Ra at any timepoint or condition (1%–6%). Cells in the CEP showed similar immunopositivity to that of the NP, between 6% and 70%. Human IL‐1β was rarely detected (0%–2%) in any condition in either the NP, AF, or CEP cells.

### 3.8 Mechano‐Transport FE Simulation Coupled With PN Equations for mRNA Expression

The mechanical simulations showed a maximum principal stress concentration at the center of the NP (Figures 9A,B) in all three models (control, IL‐1β, and IL‐1Ra). The maximum stresses were 0.91 MPa, 1.11 MPa, and 5.42 MPa for the control, IL‐1β, and IL‐1Ra‐treated groups, respectively. Glucose concentrations ranged between 2.39 and 4.91 mM (Figure 9C) while pH levels ranged between 7.06 and 7.39 within the NP for each simulated IVD (Figure 9D).

Average cell stress increased across the models, with values of 0.54 MPa (control), 0.57 MPa (IL‐1β), and 1.25 MPa (IL‐1Ra) (Table 4). In contrast, glucose and pH levels remained similar across all simulations. For direct comparison, gene expression values were obtained from the single biological replicate of each condition used to build the FE models. All three models showed a similar qualitative trend in *ACAN*, *COL2A1*, and *MMP3* expression (Figure 10). Specifically, predicted *ACAN and COL2A1* expression decreased for each statically loaded disc, corresponding to experimental gene expression decreases. Similarly, the predicted increase in *MMP‐3* expression after static loading corresponded to experimental gene expression. In contrast, predicted *COL1A1* expression was expected to increase in all three discs, while experimental gene expression only increased in the control disc. Thus, predicted *COL1A1* expression did not correspond to experimental gene expression results in discs treated with IL‐1β or IL‐1Ra. Overall, the pattern of gene expression followed the same trend as the cell stress levels.

## 4 Discussion

IVD degeneration is a highly multifactorial disease, arising from interactions among mechanical, inflammatory, and nutritional factors. Although pro‐inflammatory cytokines and abnormal loading are individually linked to catabolism, their interactions at the organ level remain unclear. We investigated whether exogenous IL‐1β alters the response of bovine IVDs to static or combined dynamic compression and torsion, and whether IL‐1Ra can mitigate loading‐induced changes. Across all groups, however, mechanical stimulation alone was sufficient to induce several degenerative readouts, while cytokine supplementation produced no additional measurable effect.

### 4.1 Mechanical Loading Dominated Tissue‐Level Responses

All mechanically stimulated IVDs showed loss of height and volume consistent with a pathological response rather than physiological diurnal changes [22, 34, 35]. Height loss exceeded the previously defined pathological threshold for bovine caudal discs in both static and dynamic loading, with the largest reductions under combined dynamic compression and torsion. Previous studies have seen similar height losses under both static loading and combined compression and torsional loading [22, 36] and have reported upregulation of catabolic cytokines such as IL‐6 and IL‐8 following mechanical overloading [37]. Under degeneration, cells of the IVD produce IL‐1β and further exacerbate the degenerative cascade; however, this was not seen in this study (see Figure S2). Furthermore, this study used non‐degenerate caudal bovine discs, which should have low levels of IL‐1 expression and thus may show a different response than what would be seen in degenerate human discs [9, 10]. Interestingly, volume loss was less pronounced than height loss, conveying that the height decrease was in parallel with an increase in width of the IVDs. These height changes occurred despite minimal differences in NP GAG or tissue‐level hydration measured post‐culture. Notably, prior investigation of combined compression and torsional loading only found a significant decrease in the GAG content of the NP under a high‐stress regime in which imposed torsion reached 15 degrees of axial rotation, while our study was programmed to a maximum of 6 degrees axial rotation as this is the highest magnitude of torsion measured in human IVDs. Additionally, when isolating the tissue for downstream analysis the amount of tissue was split for qPCR and GAG quantification, thus measurements do not necessarily represent total GAG content within each IVD, but rather the GAG content per ~50 mg of NP tissue or ~100 mg of AF tissue. MRI‐derived hydration values confirmed that water content decreased in the NP from day 0 to day 7 after dynamic loading, suggesting that rapid rehydration after unloading may partly mask biochemical measurements obtained later. Overall, structural measurements indicate that the loading regimes imposed, particularly the dynamic protocol, were supraphysiological for bovine IVDs.

Dynamic loading also induced morphological changes, including NP bulging into the endplate region consistent with Schmorl's nodes [38, 39]. This type of herniation generally occurs through endplate defects and is often asymptomatic but has been linked to Modic changes. Additionally, it has been suggested that Schmorl's nodes represent an early stage of endplate osteoporotic fracture, and singular traumatic injuries are believed to lead to Schmorl's nodes [38]. Moreover, in vivo studies in rats have shown that repetitive mechanical stress induces Schmorl's nodes formation, [40] which has likely occurred from the harsh repetitive active phase in the dynamically loaded bovine IVDs of our study. Although our study cannot definitively determine whether these defects reflect physiological failure modes or technical overloading, their consistent presence indicates that the combined torsion and compression regime exceeded the mechanical tolerance of the bovine BEP. This serendipitous finding suggests that the model may be useful for studying endplate‐driven failure mechanisms, but it also highlights that the loading regimen may not be optimal for isolating cytokine‐specific effects.

Interestingly, a higher maximum principal stress concentration was predicted at the center of the NP, adjacent to the endplate, in all three simulated bovine IVDs, indicating that this location is vulnerable under high mechanical loads. This point of weakness identified through the FE models was experimentally corroborated by the Schmorl's nodes found in the dynamically loaded IVDs. Although these dynamically loaded IVDs were not modeled, it appears that this weak point under high dynamic compression can lead to herniations when torsion is additionally imposed.

Cell metabolic activity declined across all tissues after 7 days, most profoundly in the NP, consistent with prior reports of reduced viability under prolonged loading in organ culture. AF metabolic activity decreased to a lesser degree. Overall, mechanical loading alone was sufficient to induce structural and metabolic changes typically associated with early IVD degeneration.

### 4.2 Transcript‐Level Changes Were Modest and Not Cytokine‐Specific

Relative to initial expression, anabolic marker *ACAN* decreased in the NP and CEP, while catabolic marker *MMP‐13* increased in the AF. Interestingly, protease *MMP‐3* followed a different pattern than *MMP‐13*, decreasing in the AF when there was no cytokine stimulation. However, differences among treatment groups were not statistically significant. Given the variability between donors and minor technical issues during processing, additional biological replicates should be evaluated to confirm these shifts in gene expression. Importantly, no consistent effect of IL‐1β or IL‐1Ra supplementation was detectable at the transcript level.

### 4.3 Cytokine Detection and Diffusion Remain Unresolved

Neither ELISA (see Figure S3) nor immunohistochemistry detected human IL‐1β in any tissue region. The homology of the antigen site for the IL‐1β antibody (M421B, ThermoFisher Scientific) to bovine was ~61%; however, the lack of immunopositivity found in our bovine discs suggests that there was no cross‐reactivity of the human IL‐1β antibody in bovine samples. The lack of detection may relate to the short half‐life of IL‐1β, limited diffusion through the dense ECM, or the use of a human‐specific antibody with low predicted bovine cross‐reactivity [41]. Additionally, epitope masking during prolonged decalcification may have impeded detection, and thus future studies should consider adding an additional post‐fixation step following decalcification and prior to dehydration and embedding in paraffin. Yet, it was expected that the CEP would see the most effects from IL‐1β or IL‐1Ra stimulation as the cytokines had a smaller diffusion distance needed to reach the cells than in the central NP. Thus, while the short half‐lives can in part explain the lack of change in the NP, the lack of change in the cellular response of the CEP indicates there are other contributing factors, such as the possibility that the added cytokines were negated by the natural response of the bovine IVD to the loading. As IL‐1β penetration could not be verified, we cannot conclude whether the cytokine was absent, degraded, or undetectable.

In contrast, IL‐1Ra immunopositivity was observed in NP, inner AF, and CEP regions at day 0 and in IL‐1β‐ and IL‐1Ra‐supplemented groups, suggesting antibody cross‐reactivity with bovine IL‐1Ra. The homology of the antigen site for the IL‐1Ra antibody (ab124962, abcam) to bovine was ~66%. However, only a single biological replicate was available for histological analysis, limiting the strength of these observations. Given the lack of detectable exogenous IL‐1β and the absence of an inflammatory readout, we cannot conclude whether endogenous IL‐1 signaling was activated by loading or whether IL‐1Ra counteracted any cytokine‐mediated effects. Future studies would also benefit from assessment of other direct inhibitors of IL‐1β, such as the peptide Link N [42]

Additionally, the dosage of 10 ng/mL of IL‐1Ra may have been too low to elicit any effect on IL‐1 activity [43, 44] A 10–100‐fold excess of IL‐1Ra would be required to inhibit half the activity of IL‐1 in vitro [43, 44] thus a concentration of at least 100 ng/mL of IL‐1Ra should be used in future studies. Further, the gene expression should have been checked at earlier timepoints to see changes in the cellular response to pro‐inflammatory stimulation [9] It should also be noted that histological staining revealed some remnants of the growth plate on the isolated IVDs, possibly blocking some diffusion of the cytokines as well as nutrients to the disc [14] However, it is clear that a majority of the growth plate was removed on all discs and thus nutrient transport remained feasible.

### 4.4 Finite Element Modeling Highlighted Mechanical Drivers of Cellular Response

The FE simulations, while limited to static loading due to convergence constraints, generally reproduced reductions in *ACAN* and *COL2A1* and increases in *MMP‐3* in the NP for the corresponding experimental discs. Variations in stress distribution were largely attributable to disc‐specific morphology rather than treatment. Glucose, pH, and fixed charge density remained similar across models, whereas cell stress differed markedly and aligned with predicted gene expression changes. Although these models cannot directly simulate cytokine transport or dynamic loading, they suggest that cell stress may be a stronger predictor of gene‐level responses than the biochemical environment under the tested conditions. Importantly, the FE mesh was adapted from a human model and requires recalibration for bovine tissue before quantitative interpretation.

## 5 Conclusions and Limitations

Both static and combined dynamic compression and torsion induced structural, metabolic, and transcript‐level changes consistent with early degeneration in bovine IVDs after 7 days. Under these loading regimes, exogenous IL‐1β did not produce additional measurable effects, and IL‐1Ra at 10 ng/mL did not prevent degeneration. However, cytokine diffusion and stability could not be verified, and the cross‐reactivity of human antibodies with bovine proteins limits interpretation of the protein‐level data. The absence of inflammatory readouts precludes determining whether loading activated endogenous IL‐1 signaling.

This study is further limited by the small number of biological replicates, modest RNA yield, the inability to model dynamic loading computationally, and histology from only one disc. The supraphysiological loading regime likely overshadowed cytokine‐specific effects. Future studies should incorporate lower, physiologically relevant loading magnitudes, earlier time points for molecular analysis, bovine‐specific cytokine antibodies, and quantitative assessments of inflammatory mediators. Additional biological replicates for histology and refinement of bovine‐specific FE meshes will improve the interpretation of mechanobiological interactions in organ‐level IVD culture.

## Author Contributions

Conceptualization: Katherine B. Crump, Jérôme Noailly, and Benjamin Gantenbein. Methodology: Katherine B. Crump, Paola Bermudez‐Lekerika, Christine L. Le Maitre, Jérôme Noailly, and Benjamin Gantenbein. Investigation: Katherine B. Crump. Kim de Graaf, and Estefano Muñoz‐Moya. Writing – original draft: Katherine B. Crump. Writing – review and editing: Katherine B. Crump, Estefano Muñoz‐Moya, Kim de Graaf, Paola Bermudez‐Lekerika, Christine L. Le Maitre, Jérôme Noailly, and Benjamin Gantenbein. Funding acquisition: Christine L. Le Maitre, Jérôme Noailly, and Benjamin Gantenbein. Resources: Jérôme Noailly and Benjamin Gantenbein. Supervision: Christine L. Le Maitre, Jérôme Noailly, and Benjamin Gantenbein.

## Funding

This work was supported by H2020 Marie Skłodowska‐Curie Actions, #955735.

## Conflicts of Interest

The authors declare no conflicts of interest.

## Supporting information


**Table S1:** Details of bovine discs scanned for MRI
**Figure S1:** IHC isotype controls.
**Figure S2:** qPCR of IL‐1β and ADAMTS4.
**Figure S3:** ELISA of human IL‐1β in the NP, AF, and CEP of bovine IVDs for each condition on day 0 and after 7 days of culture in the bioreactor or with static loading.

## Data Availability

Further information and requests for resources and reagents should be directed to and will be fulfilled by the corresponding author (benjamin.gantenbein@unibe.ch), Benjamin Gantenbein. This study did not generate new unique reagents. Any additional information required to reanalyze the data reported in this paper is available from the lead contact upon request.
