# Supplementary material for: Complex Mechanical Loading and Pro‐Inflammatory Cytokines in Intervertebral Disc Degeneration
Source: JOR Spine. 2026 Jan 26;9(1):e70159. doi: 10.1002/jsp2.70159 (PMC12835194; doi:10.1002/jsp2.70159)
Supplement: Supplementary file 1 — Table S1: Details of bovine discs scanned for MRI Figure S1: IHC isotype controls. Figure S2: qPCR of IL‐1β and ADAMTS4. Figure S3: ELISA of human IL‐1β in the NP, AF, and CEP of bovine IVDs for each condition on day 0 and after 7 days of culture in the bioreactor or with static loading. [file JSP2-9-e70159-s001.pdf]

Table S1. Bovine IVDs from each donor which were scanned using MRI.

	Static			Dynamic		
	Control	IL-1β	IL-1Ra	Control	IL-1β	IL-1Ra
Donor 1 (B308)	x	x		x	x	
Donor 2 (B314)		x	x	x	x	x
Donor 3 (B334)		x		x	x	x
Donor 4 (B335)			x	x	x	X
Donor 5 (B336)		x	x	x	x	x

Figure S1. *Relative gene expression of ADAMTS4 and IL-1β in the NP, AF, and CEP after culture. Data is normalized to 18S using the $2^{-\Delta Ct}$ method. All data points shown with medians. n = 1-3*

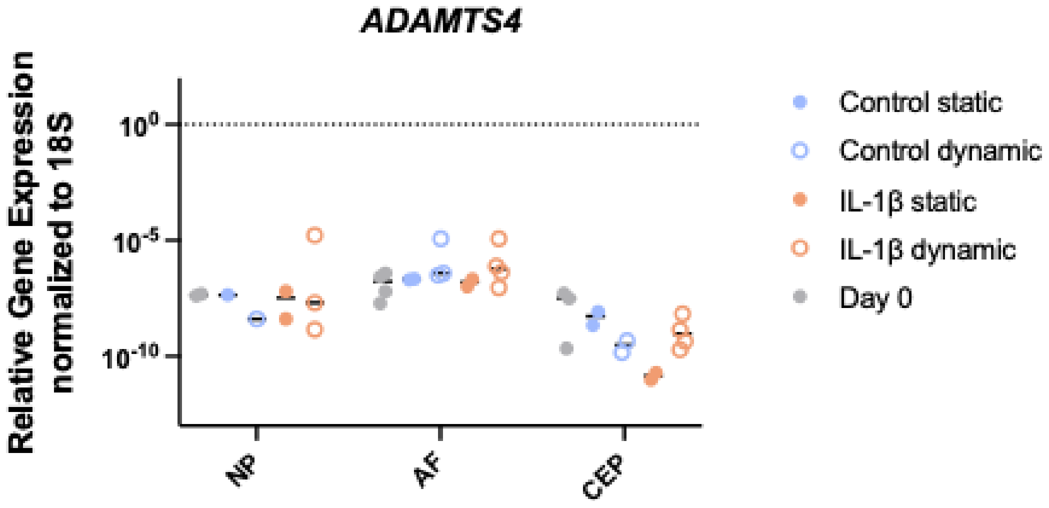

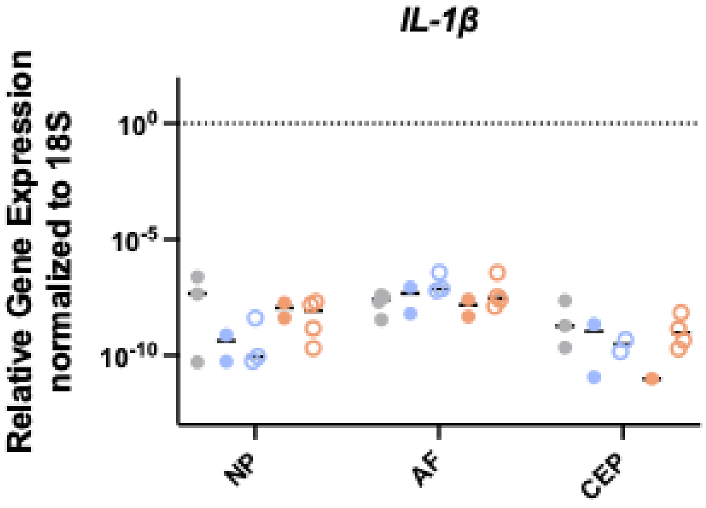

Figure S2. *Negative isotype controls for immunohistochemistry for IL-1β and IL-1Ra antibodies in bovine IVDs. Mouse IgG2b was used for IL-1β, and Rabbit IgG was used for IL-1Ra. Shown are the whole IVDs (left) and a 10X zoomed image of the endplate and nucleus pulposus (right).*

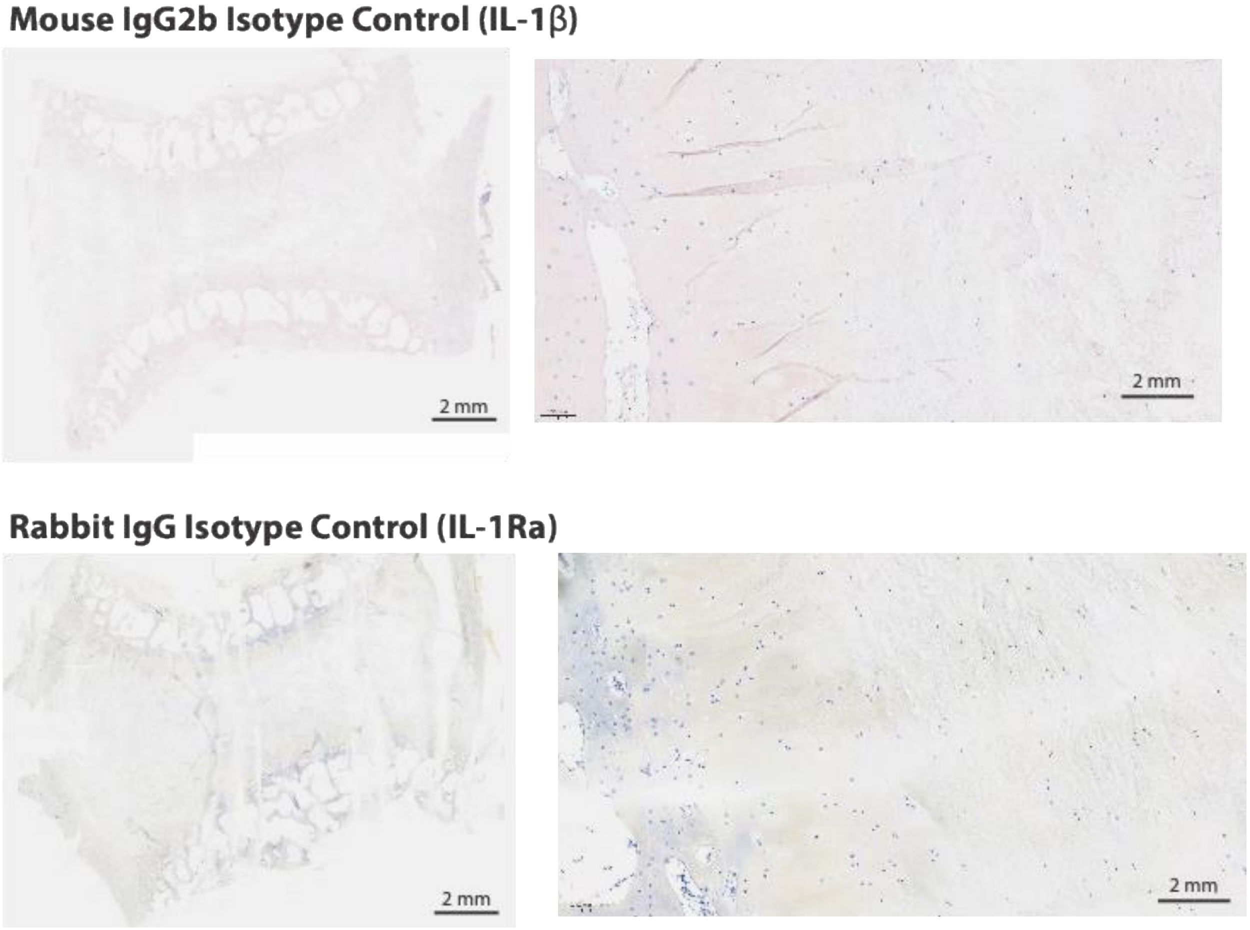

Figure S3. *ELISA of human IL-1β in the NP, AF, and CEP of bovine IVDs for each condition on day 0 and after 7 days of culture in the bioreactor or with static loading.*

Protein content of the bovine IVD samples was measured using a bicinchoninic acid assay (BCA) and then diluted with PBS to 500 ug/mL and 1000 ug/mL. Human IL-1β protein was quantified using the ELISA kit for IL-1β (SEA563Hu, Cloud-Clone Corp.). The ELISA was run according to manufacturer’s protocol and absorbance was measured using an ELISA reader (Spectramax M5, Molecular Devices, Bucher Biotec AG, Basel, Switzerland) at 450 nm.

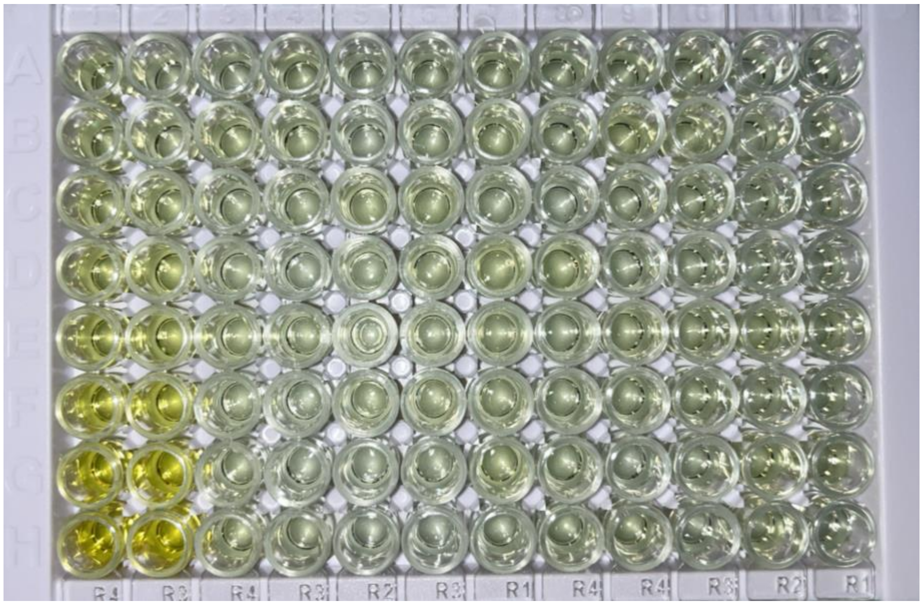

The first 2 columns on the left show the standards. Columns 3-12 are of the proteins extracted from the bovine IVD NP, AF, and CEP samples. No signal for IL-1β was detected in any bovine IVD sample.
